# A Novel Model to Predict Esophageal Varices in Patients with Compensated Cirrhosis Using Acoustic Radiation Force Impulse Elastography

**DOI:** 10.1371/journal.pone.0121009

**Published:** 2015-03-31

**Authors:** Yehyun Park, Seung Up Kim, Soo Young Park, Beom Kyung Kim, Jun Yong Park, Do Young Kim, Sang Hoon Ahn, Won Young Tak, Young Oh Kweon, Kwang-Hyub Han

**Affiliations:** 1 Department of Internal Medicine, Institute of Gastroenterology, Yonsei University College of Medicine, Seoul, Republic of Korea; 2 Liver Cirrhosis Clinical Research Center, Yonsei University College of Medicine, Seoul, Republic of Korea; 3 Kyungpook National University School of Medicine, Daegu, Republic of Korea; Taipei Veterans General Hospital, TAIWAN

## Abstract

**Background & Aims:**

Few noninvasive methods can accurately identify esophageal varices (EVs) in patients with compensated cirrhosis. We developed and validated a novel, acoustic radiation force impulse (ARFI) elastography-based prediction model for high-risk EVs (HEVs) in patients with compensated cirrhosis.

**Methods:**

A total of 143 patients with compensated cirrhosis between February, 2010 and February, 2013 (training set) and 148 between June, 2010 and May, 2013 (validation set) who underwent ARFI elastography and endoscopy were prospectively recruited. Independent predictors of HEVs were used to construct a prediction model.

**Results:**

Based on multivariate analysis, we developed two new statistical models, a varices risk score and ARFI-spleen diameter-to-platelet ratio score (ASPS), the latter of which was calculated as ARFI velocity × spleen diameter/platelet count. The area under receiver operating characteristic curve (AUROC) of the varices risk score and ASPS to predict HEVs were 0.935 (95% confidence interval [CI] 0.882–0.970) and 0.946 (95% CI 0.895–0.977), respectively. When ASPS, a simpler model with a higher AUROC, was applied in the validation set, acceptable diagnostic accuracy for HEVs was observed (AUROC = 0.814 [95% CI 0.743–0.885]). To detect HEVs, a negative predictive value of 98.3% was achieved at ASPS <2.83, whereas a positive predictive value of 100% was achieved at ASPS >5.28.

**Conclusions:**

ASPS, a novel noninvasive ARFI-based prediction model, can accurately identify HEVs in patients with compensated cirrhosis. ASPS <2.83 may safely rule out the presence of HEVs, whereas patients with ASPS >5.28 should be considered for endoscopic examinations or appropriate prophylactic treatment.

## Introduction

Esophageal varices (EVs) resulting from portal hypertension are a serious and important complication of cirrhosis. Current guidelines recommend screening endoscopy for all patients with cirrhosis to identify those who should undergo prophylactic treatment.[1] However, the majority of patients undergoing screening endoscopy either do not have varices or have varices that do not require prophylactic treatment, as the prevalence of these high-risk EVs (HEVs) at any given point in time is ~15–25%.[2]

Accordingly, noninvasive methods for diagnosing EVs in cirrhotic patients are required to avoid unnecessary invasive screening endoscopic examinations, especially in low-risk patients with cirrhosis. Although several parameters associated with the presence of EVs in compensated patients such as low platelet count,[3] large spleen diameter,[4,5] and increased Child-Pugh score[3] have been reported, none are sufficiently accurate to predict EVs when tested in independent validation series.[1,4] More recently, liver stiffness (LS) and spleen stiffness (SS) measured by transient elastography (TE) have been proposed as alternative noninvasive methods to diagnose portal hypertension and EVs.[6,7] However, TE exhibited a relatively low specificity in terms of EV prediction in one meta-analysis, although the capacity thereof to predict clinically significant portal hypertension was relatively high (sensitivity 90%, specificity 79%).[8] In addition, TE exhibits a high measurement failure rate in patients with narrow intercostal spaces, high body mass index, or ascites.[9] Another limitation is that TE is based on M-mode imaging without real-time visualization of the liver parenchyma.

In contrast, acoustic radiation force impulse (ARFI) imaging, which has been proposed as a new ultrasound-based method for noninvasive assessment of liver fibrosis, can be used even in obese patients and in those with ascites.[10,11] The success rate of ARFI measurements is higher than that of TE,[12] and enhanced accessibility due to incorporation within a conventional ultrasound system also provides ARFI with clinical advantages over TE. Although promising results regarding the accuracy of ARFI imaging for noninvasive assessment of liver fibrosis have been reported,[13,14] few studies have evaluated the utility of ARFI elastography for predicting EVs.[15–19] Thus, the aims of our study were to evaluate whether LS measured using ARFI elastography can predict the presence and severity of EVs and HEVs in patients with compensated cirrhosis, to generate a simple, ARFI-based noninvasive prediction model from a training cohort, and to validate the usefulness of this model in a validation cohort.

## Patients and Methods

This study was conducted in two parts. First, we attempted to identify variables associated with the presence of EVs and HEVs and constructed an ARFI-based prediction model for HEVs using the training set. Second, we evaluated the reproducibility of that model in the subsequent external validation set, which included patients enrolled at a different institution and time period. The study protocol was performed in accordance with the ethical guidelines of the 1975 Declaration of Helsinki. Written, informed consent was obtained from each patient. This study was approved by the institutional review board of each participating center (Severance Hospital, Yonsei University College of Medicine and Kyungpook University Hospital).

### Patients

#### Training set

We prospectively recruited 160 patients with compensated cirrhosis who underwent ARFI elastography and endoscopy at Severance Hospital, Yonsei University College of Medicine, Seoul, Korea between February, 2010 and February, 2013. Compensated cirrhosis was defined as cirrhosis evident in the absence of any previous or current known complication of portal hypertension including ascites, variceal bleeding, and/or hepatic encephalopathy.[20,21]

If histological information was not available (n = 113, 70.6%), liver cirrhosis was clinically diagnosed based on combined physical, laboratory, and radiological findings (ultrasonographic findings suggestive of cirrhosis, including a blunted, nodular liver edge accompanied by splenomegaly (>12cm)).[22,23]Exclusion criteria were: (1) no informed consent, (2) time between ARFI and endoscopy >3 months, (3) presence of hepatocellular carcinoma, (4) presence of portal vein thrombosis, (5) right-sided heart failure, (6) previous or current history of treatment for portal hypertension (splenectomy, partial splenic embolization, transjugular intrahepatic portosystemic shunt, balloon-occluded retrograde transvenous obliteration, β-blocker therapy, or endoscopic therapies), and (7) ARFI failure or unreliable measurement.

#### Validation set

The validation set was composed of consecutive patients with compensated cirrhosis who underwent ARFI elastography and endoscopy at Kyungpook University Hospital, Seoul, Korea between June, 2010 and May, 2013.

### Clinical and laboratory variables

Determination of the etiology of cirrhosis was made using standard diagnostic criteria. In patients with chronic viral hepatitis, hepatitis B virus (HBV) surface antigen plus HBV-DNA or anti-hepatitis C virus (HCV) antibodies plus HCV-RNA were present in the serum. Alcoholic liver disease was diagnosed in patients with characteristic changes in liver histology or those with consumption of at least 40 g of alcohol daily for 5 years or more.

Demographic data, including age, gender, body mass index, and other clinical/laboratory parameters were recorded for each patient at the time of ARFI measurements. Clinical parameters included a previous history of treatment for varices or portal hypertension. Laboratory parameters included serum albumin, bilirubin, prothrombin time, renal function, electrolytes, hemoglobin, hematocrit, leukocyte and platelet count, cholesterol, alanine aminotransferase (ALT), aspartate aminotransferase (AST), alkaline phosphatase, and γ-glutamyl transpeptidase. The Child-Pugh class was assessed based on these parameters.

### Imaging techniques

Standard ultrasonographic scanning of the abdomen, including measurement of maximum spleen diameter, and ARFI elastography, were performed on the same visit under fasting conditions. The spleen diameter was the bipolar diameter at the crossing point of the spleen hilum.[24] ARFI elastography was performed on each patient using a Siemens Acuson S2000 ultrasound system by expert sonographers who were blinded to the endoscopy results in each institute (all had performed >500 examinations)., LS was measured with ARFI elastography in each patient in supine position. The right lobe of the liver was accessed through the intercostal space with conventional B-mode ultrasound guidance, and a region of interest in the liver parenchyma 4–5 cm below the liver capsule and free of large blood vessels was selected. ARFI shear wave velocity was measured in meters per second (m/s). The median value of 10 valid measurements was calculated. ARFI failure was defined as zero valid shots, and unreliable measurements were defined as an interquartile range (IQR) to median value ratio greater than 30% or a success rate less than 60%. [16]

### Endoscopic evaluation and grading of EVs

Endoscopic evaluation and grading of EVs was performed by a single expert endoscopist in each institute (>1,000 examinations) who were blinded to the ARFI elastography results. EVs were classified based on the criteria for describing endoscopic findings of esophagogastric varices[25] as F_1_ (straight and small-caliber varices), F_2_ (tortuous veins forming bead-like appearance), or F_3_ (tumor-shaped varices). HEVs were defined as F_2_ to F_3_ EVs or F_1_ EVs with red color signs, according to the Baveno V criteria.[1]

### Calculation of noninvasive models

Age-spleen-platelet ratio index (ASPRI), AST-to-platelet ratio index (APRI),[26] and platelet count/spleen diameter ratio (PSR)[5] were calculated for all patients as described originally. ASPRI = age (years, <30 = 0; 30–39 = 1; 40–49 = 2; 50–59 = 3; 60–69 = 4; ≥70 = 5) + spleen diameter (cm)/platelet count (10^9^/L)×100; APRI = [(AST/upper limit of normal/platelet count (10^9^/L)]×100; PSR = [platelet count (10^9^/L)/spleen diameter (cm)]×100.

### Statistical analysis

Noninvasive models to identify EVs and HEVs were built from patients in the training set and validated in separate, independent patients in the validation set. First, univariate analysis was performed to detect candidate variables among various clinical factors to generate a new formula. Continuous variables were compared using the Student’s *t*-test or the Mann–Whitney *U* test, and categorical variables were compared using the χ^2^-test or Fisher’s exact test. Variables with *P*<0.10 in univariate analysis were included in the multivariate analysis, and factors with *P*<0.10 were finally selected as components of the new formula. To avoid the effect of collinearity, the Child-Pugh classification was not included in the multivariate analysis because it includes composite parameters. Based on these multivariate predictors, we derived a multiple fractional equation for prediction of EVs and HEVs.

The relationship and comparison between LS values and the severity of EVs was assessed using the Spearman correlation coefficient and Kruskal-Wallis test. Post-hoc analysis was performed using the Dunn procedure to evaluate differences between groups. The discriminative ability of the different noninvasive methods and models for identification of EVs and HEVs was assessed with receiver operating characteristic (ROC) curve analysis and expressed as the area under the ROC curve (AUROC). Comparison between AUROCs was made using the DeLong test. Sensitivity, specificity, positive predictive value (PPV), negative predictive value (NPV), positive likelihood ratio (+LR), and negative likelihood ratio (−LR) were calculated using ROC curves. Optimal cutoff values were chosen to confidently rule out and rule in patients with HEVs. Thereafter, in a subsequent validation set, we tested the diagnostic value of the formula that was derived from the training set.

*P*<0.05 was considered statistically significant. Data were analyzed using SPSS 20.0 for Windows (SPSS Inc., Chicago, IL) and MedCalc Software (version 12.7.2, MedCalc Software bvba, Ostend, Belgium).

## Results

### Selection of study subjects

**Fig 1:**
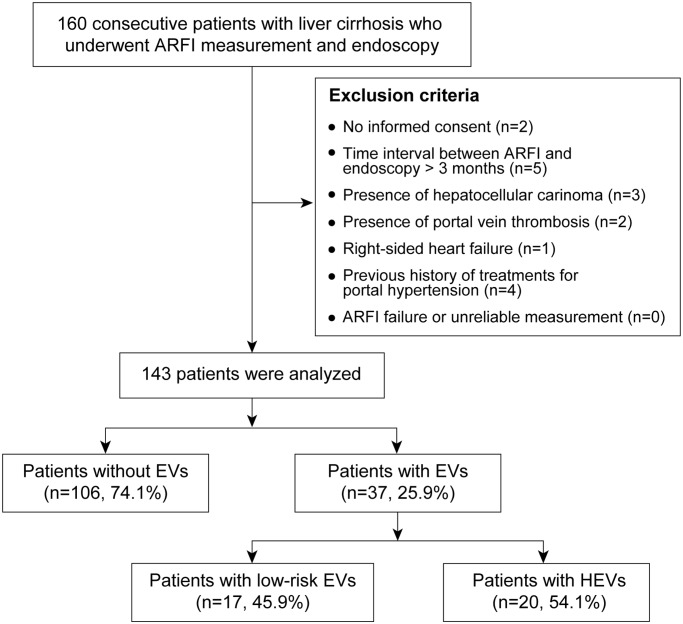
Flow of selecting study population in training set. A total of 160 consecutive patients with compensated cirrhosis were enrolled. Of these, 17 patients were excluded based on our exclusion criteria. Finally, a total of 143 patients were selected for statistical analysis. ARFI, acoustic radiation force impulse; EVs, esophageal varices; HEVs, high-risk esophageal varices.

Among 160 patients enrolled in the training set, 17 patients were excluded (Fig 1): (1) no informed consent (n = 2), (2) time between ARFI and endoscopy >3 months (n = 5), (3) presence of hepatocellular carcinoma (n = 3), (4) presence of portal vein thrombosis (n = 2), (5) right-sided heart failure (n = 1), (6) previous or current history of treatment for portal hypertension (n = 4), and (7) ARFI failure or unreliable measurement (n = 0). The remaining 143 patients were selected for the training set. The median interval between ARFI elastography and endoscopy was 4 (range, 0–90) days.

After excluding 21 patients according to the same exclusion criteria as used for the training set, the validation set was composed of 148 patients with compensated cirrhosis (13 with biopsy-proven cirrhosis and 135 with clinically diagnosed cirrhosis).

### Baseline characteristics of the training and validation sets

The clinical, biochemical, and endoscopic characteristics of the patients in the training and validation sets are summarized in Table 1. Among 143 patients in the training set (mean age 54.7 years, 82 males), most patients (n = 123, 86.0%) were classified as Child-Pugh class A. Thirty-seven (25.9%) patients had EVs, and 19 (13.3%) had HEVs.

**Table 1:** Baseline characteristics of the study population.

Characteristics	Training set (n = 143)	Validation set (n = 148)	*P* value
Demographic data			
Age (years)	55.0 (48.0–62.0)	58.0 (49.0–66.0)	0.051
Male gender	82 (57.3)	100 (67.6)	0.072
Body mass index (kg/m^2^)	24.3 (21.6–26.0)	23.1 (20.7–25.6)	0.089
Laboratory data			
Serum albumin (g/dL)	4.1 (3.6–4.3)	4.1 (3.6–4.3)	0.125
Total bilirubin (mg/dL)	0.8 (0.6–1.2)	0.8 (0.6–1.2)	0.658
Aspartate aminotransferase (IU/L)	32.0 (24.0–52.0)	37.0 (26–47)	0.258
Alanine aminotransferase (IU/L)	26.0 (16.0–44.0)	23.0 (14–36)	0.016
Prothrombin time (%)	100.0 (90.0–100.0)	79.5 (63.0–90.4)	<0.001
Platelet count (x10^9^/L)	156.0 (110.0–207.0)	141.5 (83.8–184.0)	0.114
Child-Pugh class, A/ B	123 (86.0) / 20 (14.0)	145 (98.0) / 3 (2.0)	<0.001
Etiology, HBV/ HCV/ others	88 (61.5) / 10 (7.0) / 45 (31.5)	91 (61.5) / 14 (9.5) / 43 (29.0)	0.001
Esophageal varix	37 (25.9)	51 (34.5)	0.135
F1/ F2/ F3	19 (51.3) / 13 (35.1) / 5 (13.5)	21 (41.2) / 15 (29.4) / 15 (29.4)	
High-risk esophageal varix	20 (14.0)	30 (20.3)	0.155
Portal hypertensive gastritis	10 (7.0)	20 (13.5)	0.067
ARFI velocity (m/s)	1.66 (1.26–2.28)	1.78 (1.39–2.54)	0.036
Spleen diameter (cm)	10.2 (9.1–11.7)	10.0 (9.0–12.8)	0.833

Variables are expressed as median (interquartile range) or n (%).

NS, not significant; HBV, hepatitis B virus; HCV, hepatitis C virus; ARFI, acoustic radiation force impulse.

In the validation set (n = 148, mean age 57.3 years, 101 males), 51 (34.5%) patients had EVs, and 30 (20.3%) had HEVs. Patients in the validation set had a higher ARFI velocity than those in the training set (mean 1.98 vs. 1.81 m/s, *P* = 0.036). In addition, ALT, prothrombin time, the proportion in each Child-Pugh class, and the distribution of the etiology of liver diseases were significantly different between the training and validation sets (all *P*<0.05).

### Relationship between ARFI velocity and EV severity

The mean ARFI velocities in patients with no EVs (NEVs), low-risk EVs, and HEVs were 1.64±0.57 m/s, 2.19±0.73 m/s, and 2.39±0.67 m/s, respectively (S1 Fig). The ARFI velocity increased with EV severity (correlation coefficient = 0.415; *P*<0.001) and was significantly different among the three groups (*P*<0.001). Post-hoc analysis revealed that ARFI velocities were significantly different between patients with NEVs and EVs (*P*<0.001), but similar between patients with low-risk EVs and HEVs (*P* = 0.377). When the proportion of patients with EVs and HEVs was calculated according to the stratified ARFI velocity, the prevalence of EVs and HEVs tended to increase as the ARFI velocity increased (S2 Fig).

### Correlation between ARFI velocity and other variables

Correlation analysis between the ARFI velocity and other variables is shown in S1 Table. Serum albumin, prothrombin time, AST, spleen diameter, platelet count, total bilirubin, and age were significantly correlated with the ARFI velocity (all *P*<0.05), whereas gender, BMI, and ALT were not (all *P*>0.05).

### Comparison between patients without EVs and those with EVs/HEVs in the training set

Table 2 shows the comparison between the variables for patients without EVs and those with EVs/HEVs in the training set. Univariate analysis showed that patients with EVs had significantly higher ARFI velocities and spleen diameter and lower serum albumin, prothrombin time, and platelet count compared with those with NEVs (all *P*<0.05). The proportion with Child-Pugh class A was significantly lower in patients with EVs than in those with NEVs (*P* = 0.001). The same variables were significantly different between patients with and without HEVs with univariate analysis (all *P*<0.05).

**Table 2:** Comparison between patients without EV and those with EVs/HEVs and independent predictors of EV and HEV in training set.

Characteristics	Patients with NEVs (n = 106, 74.1%)	Patients with EVs (n = 37, 25.9%)	Univariate	Multivariate	
			*P* value	Odd ratio (95% CI)	*P* value
EV					
Demographic data					
Age (years)	55.0 (46.5–61.0)	56.0 (48.5–64.5)	0.304	1.04 (0.98–1.10)	0.217
Male gender	58 (54.7)	24 (64.9)	0.283	0.88 (0.28–2.80)	0.834
Body mass index (kg/m^2^)	24.2 (21.5–25.8)	24.3 (22.1–26.8)	0.982		
Laboratory data					
Serum albumin (g/dL)	4.2 (3.8–4.4)	3.8 (3.1–4.2)	0.002	1.55 (0.53–4.61)	0.426
Total bilirubin (mg/dL)	0.7 (0.6–1.1)	1.2 (0.8–2.1)	0.585		
Alanine aminotransferase (IU/L)	29.0 (17.8–46.3)	21.0 (13.5–42.5)	0.171		
Prothrombin time (%)	100.0 (92.0–100.0)	90.0 (77.0–100.0)	<0.001	0.98 (0.93–1.03)	0.357
Platelet count (x10^9^/L)	174.5 (130.8–219.0)	84.0 (60.5–127.5)	<0.001	0.98 (0.97–0.99)	<0.001
Child-Pugh class A	97 (91.5)	26 (70.3)	0.002		
Viral etiology	71 (67.0)	27 (73.0)	0.500		
Spleen diameter (cm)	10.0 (8.8–10.9)	12.8 (10.3–14.1)	<0.001	1.30 (0.97–1.75)	0.076
ARFI velocity (m/s)	1.51 (1.19–2.03)	2.40 (1.69–2.66)	<0.001	4.30 (1.73–10.70)	0.002
HEV					
Demographic data					
Age (years)	55.0 (48.0–62.0)	52.5 (45.8–61.5)	0.941	0.97 (0.90–1.05)	0.431
Male gender	71 (57.3)	11 (57.9)	0.796	1.79 (0.38–8.33)	0.459
Body mass index (kg/m^2^)	24.2 (21.3–25.8)	24.7 (23.0–27.2)	0.252		
Laboratory data					
Serum albumin (g/dL)	4.2 (3.7–4.4)	3.5 (2.9–4.0)	<0.001	0.51 (0.15–1.69)	0.270
Total bilirubin (mg/dL)	0.8 (0.6–1.1)	1.3 (1.0–2.2)	0.716		
Alanine aminotransferase (IU/L)	26.0 (15–44)	29.5 (17–46.3)	0.566		
Prothrombin time (%)	100.0 (91.0–100.0)	86.0 (73.5–96.3)	<0.001	1.00 (0.96–1.09)	0.475
Platelet count (x10^9^/L)	166.0 (126.0–214.0)	65.5 (58.3–93.0)	<0.001	0.97 (0.95–0.99)	0.005
Child-Pugh class A	112 (90.3)	11 (57.9)	0.001		
Viral etiology	83 (66.9)	15 (78.9)	0.241		
Spleen diameter (cm)	10.1 (8.9–11.0)	13.1 (12.0–14.4)	<0.001	1.41 (0.98–2.04)	0.068
ARFI velocity (m/s)	1.54 (1.23–2.06)	2.41 (2.05–2.62)	<0.001	4.32 (1.38–13.54)	0.012

Variables are expressed as median (interquartile range).

EVs, esophageal varices; HEV, high-risk esophageal varices; NEV, no esophageal varices; CI, confidence interval; ARFI, acoustic radiation force impulse.

### Independent predictors of EVs and HEVs and development of the EV prediction formula in the training set

Multivariate logistic regression analysis using the seven variables (age, gender, serum albumin, prothrombin time, platelet count, spleen diameter, and ARFI velocity) that were significant in univariate analysis showed that platelet count (*P*<0.001; odds ratio 0.98; 95% confidence interval [CI] 0.965–0.990) and ARFI velocity (*P* = 0.002; odds ratio 4.30; 95% CI 1.727–10.700) were independent parameters associated with the presence of EVs (Table 2). These two factors were selected as independent predictors of HEVs in multivariate analysis (all *P*<0.05; Table 2).

The spleen diameter, which showed borderline statistical significance (*P* = 0.076), was also incorporated into the final model to develop the EV prediction formula in the training set.

The equation for the model (***varices risk score***) is:

$$varices\;risk\;score=-3.604+\left(1.465\times ARFI\;velocity\right)+\left(0.247\times spleen\;diameter\;\left[cm\right]\right)-\left(0.023\times platelet\;count\left[\times {\text{10}}^{\text{9}}\;\text{/L}\right]\right)$$.

To generate simpler model easy to use in clinical practice, we also derived a multiple fractional equation for predicting EVs based on multivariate analysis. This simpler model included ARFI velocity (odds ratio>1.0) and spleen diameter (odds ratio>1.0) as the numerator, and platelet count (odds ratio <1.0) as the denominator, to amplify the effect of each factor for predicting EVs and was named as ***ARFI-spleen diameter to platelet ratio score (ASPS)***: ARFI velocity (m/s)×spleen diameter (mm)/platelet count (×10^9^/L). Although the spleen diameter only showed borderline statistical significance (*P* = 0.076), it was incorporated into the ASPS model due to its significance in predicting EVs in previous studies.[2,26]

### Diagnostic performances of the varices risk score, ASPS, and other noninvasive models to predict EVs and HEVs

The varices risk score (AUROC = 0.906 for EVs; 0.935 for HEVs) and ASPS (AUROC = 0.903 for EVs; 0.946 for HEVs) performed better than the other noninvasive models for diagnosing EVs and HEVs in the training set (S3 Fig, Table 3). Both the varices risk score and ASPS were clearly superior to ARFI velocity alone (AUROC = 0.769 for EVs; 0.777 for HEVs) for diagnosing EVs and HEVs (both *P*<0.05). Similar results were obtained when only Child-Pugh class A patients (123 patients in the training set, 145 patients in the validation set) were analyzed; the accuracy of diagnosis of HEVs with ASPS was 93.5% (S2 Table). Also, when we compared ARFI velocity/platelet counts with ASPS data, the latter modality afforded significantly better performance in terms of prediction of both EVs and HEVs (AUROCs 0.903 vs. 0.861 for EVs, *P* = 0.026; AUROCs 0.946 vs. 0.905 for HEVs, *P* = 0.006, by the DeLong method). Because no statistical difference between AUROCs of the varices risk score and ASPS for detecting EVs and HEVs (both *P*>0.05) was found, ASPS, a simpler method, was used for further statistical analysis.

**Table 3:** Diagnostic performances of non-invasive models for prediction of EVs and HEVs in the training set.

	Method	AUROC (95% CI)	Cutoff value	Sensitivity (%)	Specificity (%)	PPV (%)	NPV (%)	+LR	-LR	Accuracy (%)
EVs	Varices risk score	0.906 (0.845–0.948)	-0.95	81.1	84.9	65.2	92.8	5.37	0.22	83.9
	ASPS	0.903 (0.847–0.960)	1.67	81.1	84.0	63.8	92.7	5.06	0.23	83.2
	ASPRI	0.887 (0.830–0.944)	10.3	89.2	74.5	55.0	95.2	3.50	0.15	77.6
	PSR	0.878 (0.819–0.937)	1357.7	86.5	73.6	53.3	94.0	3.27	0.18	76.9
	APRI	0.772 (0.689–0.856)	0.74	81.1	66.0	45.5	90.9	2.39	0.29	69.2
	ARFI	0.769 (0.680–0.858)	2.08	64.9	81.1	54.5	86.9	3.44	0.43	76.9
HEVs	Varices risk score	0.935 (0.882–0.970)	0.01	90.0	89.4	58.1	98.2	8.52	0.11	89.5
	ASPS	0.946 (0.895–0.977)	2.83	90.0	94.3	72.0	98.3	15.81	0.11	93.7
	ASPRI	0.927 (0.871–0.964)	13.4	95.0	85.4	51.4	99.1	6.49	0.06	86.7
	PSR	0.920 (0.863–0.959)	899.2	90.0	87.0	52.9	98.2	6.92	0.11	87.4
	APRI	0.856 (0.787–0.909)	0.96	95.0	74.8	38.0	98.9	3.77	0.07	76.9
	ARFI	0.786 (0.709–0.850)	1.90	85.0	67.5	29.8	96.5	2.61	0.22	69.9

PPV, positive predictive value; NPV, negative predictive value; LR, likelihood ratio; EVs, esophageal varices; HEVs, high-risk esophageal varices; ASPS, ARFI-spleen diameter to platelet ratio; ASPRI, age-spleen-to-platelet ratio index; PSR, platelet-spleen ratio; APRI, AST-to-platelet ratio index; ARFI, acoustic radiation force impulse.

Regarding the validation set, the AUROCs of ASPS were 0.878 for EVs and 0.814 for HEVs, showing superiority of diagnostic accuracy over the ARFI velocity alone (AUROC = 0.795, *P* = 0.020 for EVs; 0.713, *P* = 0.044 for HEVs).

### Diagnostic performances of ASPS for predicting EVs and HEVs using dichotomic cutoffs

A single dichotomic ASPS cutoff value with both sensitivity and specificity >80% was set as 1.67 for predicting EVs and 2.83 for predicting HEVs (S3 Table). In the training set, using 1.67 as a cutoff value for EVs, 119 of 143 (83.2%) patients were correctly classified (30 as having EVs; 89 as not having EVs), whereas 24 (16.8%) were misclassified (17 false-positive; 7 false-negative). Using 2.83 as a cutoff value for HEVs, 134 of 143 (93.7%) patients were correctly classified (18 as having HEVs; 116 as not having HEVs), whereas 9 (6.3%) patients were misclassified (7 false-positive; 2 false-negative).

Results from the validation set confirmed the high discriminative power of ASPS for predicting EVs and HEVs. In the validation set, 83.1% and 76.4% patients were correctly classified using 1.67 cutoff for EVs and 2.83 cutoff for HEVs respectively.

In consideration of the most common etiology of HBV in our population, we performed subgroup analysis on patients with HBV etiologies (n = 88, 61.5% of the training set and n = 91, 61.5% of the validation set), and the results did not differ from those obtained using the entire population (S3 Table).

### Determining ASPS cutoff values for detecting and excluding HEVs

To determine two cutoffs to rule in and rule out HEVs in the training set, various cutoffs with different diagnostic indices including 95% sensitivity, 95% specificity, 95% NPV, and 95% PPV were evaluated (S4 Table). A cutoff value of ASPS 2.83 to rule out HEVs (NPV 98.3%) correctly identified the absence of HEVs in 116 of 118 patients with ASPS <2.83. Similarly, an ASPS cutoff value of 5.28 to rule in HEVs (PPV 88.9%) correctly identified the presence of HEVs in eight of nine patients with ASPS >5.28. Overall, HEVs were correctly identified in 124 of 143 (86.7%) patients.

In the validation set, using a cutoff value of ASPS 2.83, a NPV of 90.3% (93 of 103 patients with ASPS <2.83) to rule out HEVs was achieved, whereas a PPV of 60.0% (9 of 15 patients with ASPS >5.28) was obtained to identify the presence of HEVs using a cutoff value of ASPS 5.28. Overall, HEVs were correctly identified in 102 of 148 (68.9%) patients (S5 Table).

**Fig 2:**
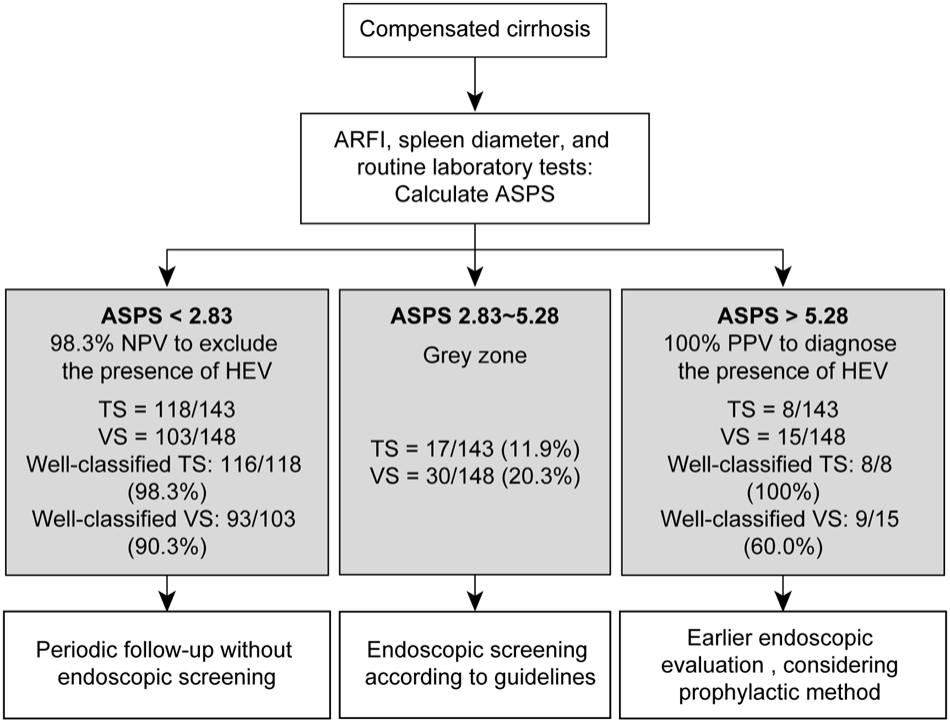
Proposed management algorithm for screening and surveillance of HEV in patients with cirrhosis. HEVs, high-risk esophageal varices; ARFI, acoustic radiation force impulse; ASPS, ARFI-spleen diameter to platelet ratio.

We constructed a novel management algorithm for HEVs in patients with compensated cirrhosis (Fig 2).

## Discussion

Recently, the availability of noninvasive methods for assessing liver fibrosis has enabled diagnosis of cirrhosis in the early, compensated stage. Despite being completely asymptomatic, these patients are at risk for decompensation and require periodic endoscopic screening for EVs and appropriate prophylactic treatment for HEVs. However, if a noninvasive screening tool is available that restricts endoscopy to selected high-risk patients, many low-risk patients could avoid or delay unnecessary endoscopy.

Here, we developed a novel, ARFI elastography-based prediction model (ASPS) for diagnosing HEVs in patients with compensated cirrhosis. ASPS showed acceptable diagnostic performances, and we validated the model in a different cohort. The AUROC of the varices risk score and ASPS to predict HEVs were significantly high (0.935 and 0.946, respectively), and when using ASPS cutoffs of 2.83 and 5.28 to rule out and rule in the presence of HEV, the percentage of correctly classified patients was 86.7% in the training set and 68.9% in the validation set. We also proposed a management algorithm based on these ASPS cutoffs.

Similar to our study, an increasing interest exists for developing noninvasive methods to predict EVs. Recently, predicting the presence of EVs and HEVs with TE or ARFI elastography has been reported in several studies.[7,15–18,27] However, the diagnostic performances differed greatly among the studies; AUROC for the presence of HEVs with TE ranged from 0.53 to 0.83[7,15,27] and that with ARFI elastography ranged from 0.60 to 0.87.[15,17,18] ARFI may have some advantages over TE for staging of liver fibrosis and predicting EVs, because TE measurement is limited in patients with ascites, obesity, and narrow intercostal spaces, leading to a rate of unsuccessful measurement of up to 18.9%,[9] compared with 2.9% in ARFI elastography, which is not limited by these conditions (no measurement failure or unreliable measurement in our study).[10] Despite this advantage of ARFI elastography, whether ARFI elastography has comparable diagnostic accuracy with TE for diagnosing EVs and HEVs is unclear, although previous studies and meta-analyses have shown comparable diagnostic accuracy for the noninvasive staging of liver fibrosis and cirrhosis with both ARFI elastography and TE.[13,14]

Because the diagnostic accuracy of ARFI alone for predicting EVs and HEVs (AUROC = 0.769 and 0.777, respectively) was unsatisfactory in our study, similar to previous studies,[15–17] we developed a highly accurate novel prediction model using three variables; the varices risk score using logistic regression model and ASPS using multiple fractional equation. These two models had statistically similar diagnostic accuracies (*P*>0.05) and showed consistently higher AUROC than those of other noninvasive models such as ASPRI, PSR, and APRI. Using the varices risk score and ASPS, we obtained a significantly higher AUROC (>0.9) over ARFI alone (both *P*<0.05). Similar to ASPS, a TE-based EV or HEV prediction model called the liver stiffness measurement-spleen diameter to platelet ratio (LSPS) index is available.[2] This index uses LS values from TE instead of ARFI elastography. The cross-sectional diagnostic accuracy of the LSPS index to predict EVs and HEVs is significantly high, and its predictive value for future variceal bleeding has been confirmed.[2,24,28] Thus, further studies and external validations are required to determine whether ASPS can predict the long-term risk of variceal bleeding from a longitudinal perspective.

Despite previous studies evaluating ARFI elastography for predicting EVs, our study has several unique features. First, we developed a simple-to-use, ARFI-based prediction model with a high AUROC (ASPS) in combination with other parameters reflecting portal hypertension. Although Bota et al.[17] previously proposed an ARFI-based prediction model for significant EVs by combining LS and SS measured with ARFI, the AUROC of their model was relatively low (0.721) with a complicated calculation formula. In contrast, ASPS is composed of three variables that are readily obtained in clinical practices and is easy to calculate with excellent diagnostic accuracy. Second, one of our aims in this study was prediction of HEVs, because the decision of whether and when prophylactic treatment should be initiated is an important clinical issue. Based on our proposed management algorithm, we were able to identify not only who should be screened endoscopically for EVs but also who should be considered for prophylactic treatment. Third, we limited the study population to ‘compensated’ cirrhotic patients, because these patients are those whom prediction of EVs is most important and efficient. Finally, using the optimal cutoff ASPS value, the presence of HEVs can be confidently ruled out in most patients with compensated cirrhosis, reducing the medical workload and financial burden to patients.

Exclusion of HEVs could be identified with a high NPV of 98.3% at a cutoff value of ASPS 2.83 in the training set, so it can be confidently applied without a significant risk of missing important diagnosis of HEVs. Using this cutoff value, 116 (81.1%) patients in the training set would avoid endoscopy, and only periodic follow-up with this noninvasive tool may be sufficient for these low-risk patients. Likewise, the presence of HEVs could be predicted with a high PPV of 88.9% at a cutoff value of ASPS 5.28. In patients with ASPS>5.28, early endoscopic examination for both diagnostic and therapeutic reasons, and appropriate commencement of β-blocker therapy, should be considered. Patients in the border zone (2.83≤ASPS≤5.28; 17 patients, 11.9%) should undergo screening endoscopy according to current guidelines. Our proposed algorithm may allow physicians to make clinical decisions about diagnosis, prophylaxis, and surveillance of EVs or HEVs. Based on our proposed algorithm, endoscopy could be safely avoided in 81.1–88.8% of patients, depending on physicians’ decisions.

Chronic HBV infection was most common both in the training and validation set (61.5%). Previous reports have suggested that performing TE to diagnose EVs differs according to the etiologies and that TE may be less accurate for predicting HEVs in liver diseases caused by factors other than HCV.[7,29] In addition, a meta-analysis by Friedrich-Rust et al.[10] concluded that diagnostic performance of ARFI elastography in patients with chronic HBV infection is lower than that in those with chronic HCV infection (AUROC 0.79 vs. 0.88 for predicting significant fibrosis [≥Metavir F_2_]). Similarly, Ye et al.[19] showed no significant correlation between LS by ARFI elastography and the severity of varices in patients with chronic HBV infection. However, despite the predominance of patients with chronic HBV infection, ASPS showed excellent performance in diagnosing HEVs (AUROC 0.935), and subgroup analysis with patients with HBV infection also showed good diagnostic performance (AUROC = 0.929 in the training set; 0.838 in the validation set, data not shown). Thus, the performance of ARFI or ASPS to predict EVs or HEVs in patients with HBV infection should be examined further in future studies.

Several issues remain unresolved in our study. First, because we used a cross-sectional study design, the performance of ASPS to predict future development of HEVs or EVs using serial measurements requires further evaluation in longitudinal studies. Second, if the hepatic venous pressure gradient, which is obtained with an invasive procedure but the gold standard for diagnosing portal hypertension, had been measured, the quantitative analysis of various parameters including ARFI elastography may have been better established. Third, despite the excellent NPV for excluding HEVs, ASPS had a relatively lower PPV, indicating that unnecessary endoscopies will be performed in patients without HEVs. Although the exact reason for this phenomenon is not clear, the reason is probably partially due to a relatively low prevalence of HEVs in our population. Finally, SS was not measured in this study. In recent studies, the relationship between SS measured by ARFI elastography and the presence of EVs and HEVs has been assessed. Takuma et al.[16] reported the high diagnostic performance of SS for the presence of HEVs (AUROC = 0.930). In contrast, Vermehren et al.[15] reported that the diagnostic performance of SS for predicting large EVs was significantly low (AUROC = 0.58), and Rifai et al.[30] also showed that SS is inferior to LS with ARFI elastography for detecting portal hypertension (AUROC 0.68 vs. 0.90). Thus, the diagnostic value of SS with ARFI elastography to predict EVs or portal hypertension remains controversial. In the present study, spleen diameter was a component of our predictive model. Spleen diameter is often considered to be a measure of portal hypertension. In studies on noninvasive parameters useful to predict EVs, spleen diameter was evaluated either alone or in combination with other parameters.[2,4,5,24,26,28] When used alone, spleen diameter is of limited utility in predicting either EVs or portal hypertension; Takuma et al.[16] showed that SS was a better predictor of EVs than spleen diameter. However, when spleen diameter is used as a component of an EV-predictive model, the performance thereof was much enhanced. In the present study, an ASPS incorporating spleen diameter afforded the best performance of several non-invasive models. When it is considered that splenomegaly can also be caused by hyperactivation of splenic lymphoid tissue and changes in hyperdynamic circulatory status, SS (which reflects hyperdynamic status) may be a better measure of portal hypertension. However, SS requires external validation, and further studies comparing SS with combinations of LS, platelet count, and spleen diameter, would be worthwhile. Also, future global multi-center study is required to validate the utility of the ASPS in both non-Asian and Asian populations.

In conclusion, ASPS, a novel, simple-to-use ARFI-based noninvasive prediction model for HEVs, was developed in this prospective study. ASPS showed excellent diagnostic performance for predicting HEVs in patients with compensated cirrhosis, and external validation confirmed the usefulness of this new formula in independent populations. ASPS <2.83 may safely rule out the presence of HEVs, whereas patients with ASPS >5.28 should be considered for endoscopic examination and appropriate prophylactic treatment if indicated. These results may lead to a reduction in the number of unnecessary endoscopies in patients with compensated cirrhosis.

## Supporting Information

S1 Fig Distribution of ARFI velocity in patients with NEV, low-risk EV, and HEVs. Each *dot* represents a patient, and *bars* indicate mean values. Median ARFI velocities were 1.51 m/s, 2.28 m/s, and 2.40 m/s for NEV, low-risk EV, and HEV respectively. ARFI, acoustic radiation force impulse; NEV, no esophageal varices; EVs, esophageal varices; HEVs, high-risk esophageal varices.
(JPG)

S2 Fig Prevalence of EV (A) and HEV (B) according to stratified ARFI velocity. The prevalence of EVs and HEVs tends to increase as ARFI velocity increases.
(TIF)

S3 Fig ROC curves of ARFI-based varices risk score, ASPS, ASPRI, PSR, APRI, and ARFI velocity for the diagnosis of EVs (A) and HEVs (B) in the training set. Varices risk score and ASPS showed the best diagnostic performance in predicting EVs and HEVs (AUROC = 0.906 and 0.903 for EVs; 0.935 and 0.946 for HEVs, respectively; all *P*<0.05). AUROCs are given in brackets after each non-invasive model. ASPS, ARFI-spleen diameter to platelet ratio; ASPRI, age-spleen-to-platelet ratio index; PSR, platelet-spleen ratio; APRI, AST-to-platelet ratio index; ARFI, acoustic radiation force impulse; EVs, esophageal varices; HEV, high-risk esophageal varices
(TIF)

S1 Table Correlation analysis between ARFI velocity and other variables. (DOCX)

S2 Table Diagnostic performances of non-invasive models for prediction of EVs and HEVs in the training set among patients with Child-Pugh class A (n = 123 patients in the training set). (DOCX)

S3 Table Diagnostic performances of ASPS for prediction of EVs and HEVs in the training and validation sets using dichotomic cutoffs in the entire population and subgroup with HBV. (DOCX)

S4 Table Diagnostic performances of ASPS cutoffs for prediction of HEVs in the training and validation sets. (DOCX)

S5 Table Diagnostic performances of the chosen cutoffs of ASPS for predicting HEVs. Performance of the use of two cutoffs (one to rule out EVs, and one to rule in EVs).
(DOCX)
