# Supplementary figures and images for: A Novel Model to Predict Esophageal Varices in Patients with Compensated Cirrhosis Using Acoustic Radiation Force Impulse Elastography
Source: PLoS One. 2015 Mar 31;10(3):e0121009. doi: 10.1371/journal.pone.0121009 (PMC4380431; doi:10.1371/journal.pone.0121009)

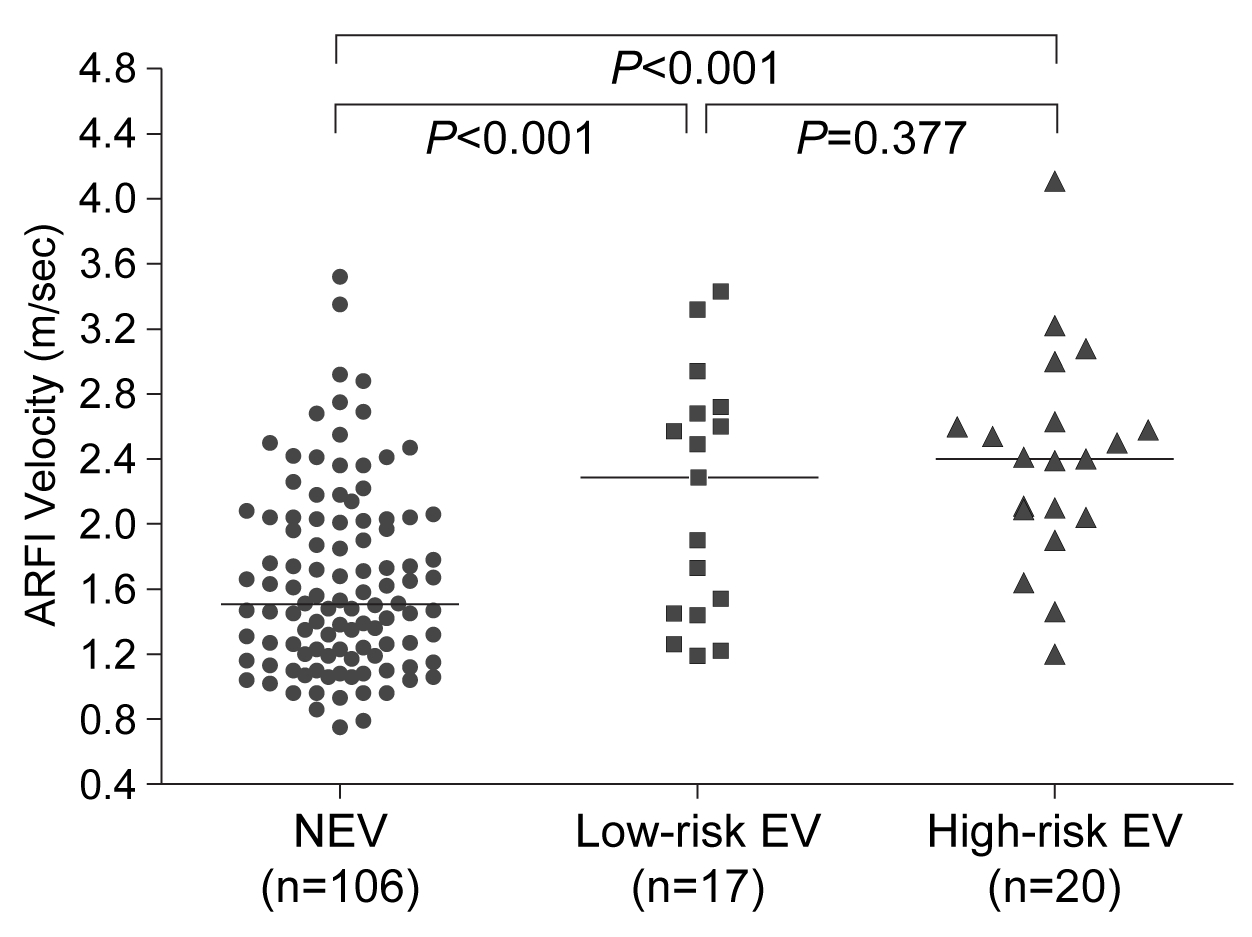

Supplement: S1 Fig — Each dot represents a patient, and bars indicate mean values. Median ARFI velocities were 1.51 m/s, 2.28 m/s, and 2.40 m/s for NEV, low-risk EV, and HEV respectively. ARFI, acoustic radiation force impulse; NEV, no esophageal varices; EVs, esophageal varices; HEVs, high-risk esophageal varices. (JPG) [file pone.0121009.s001.jpg]

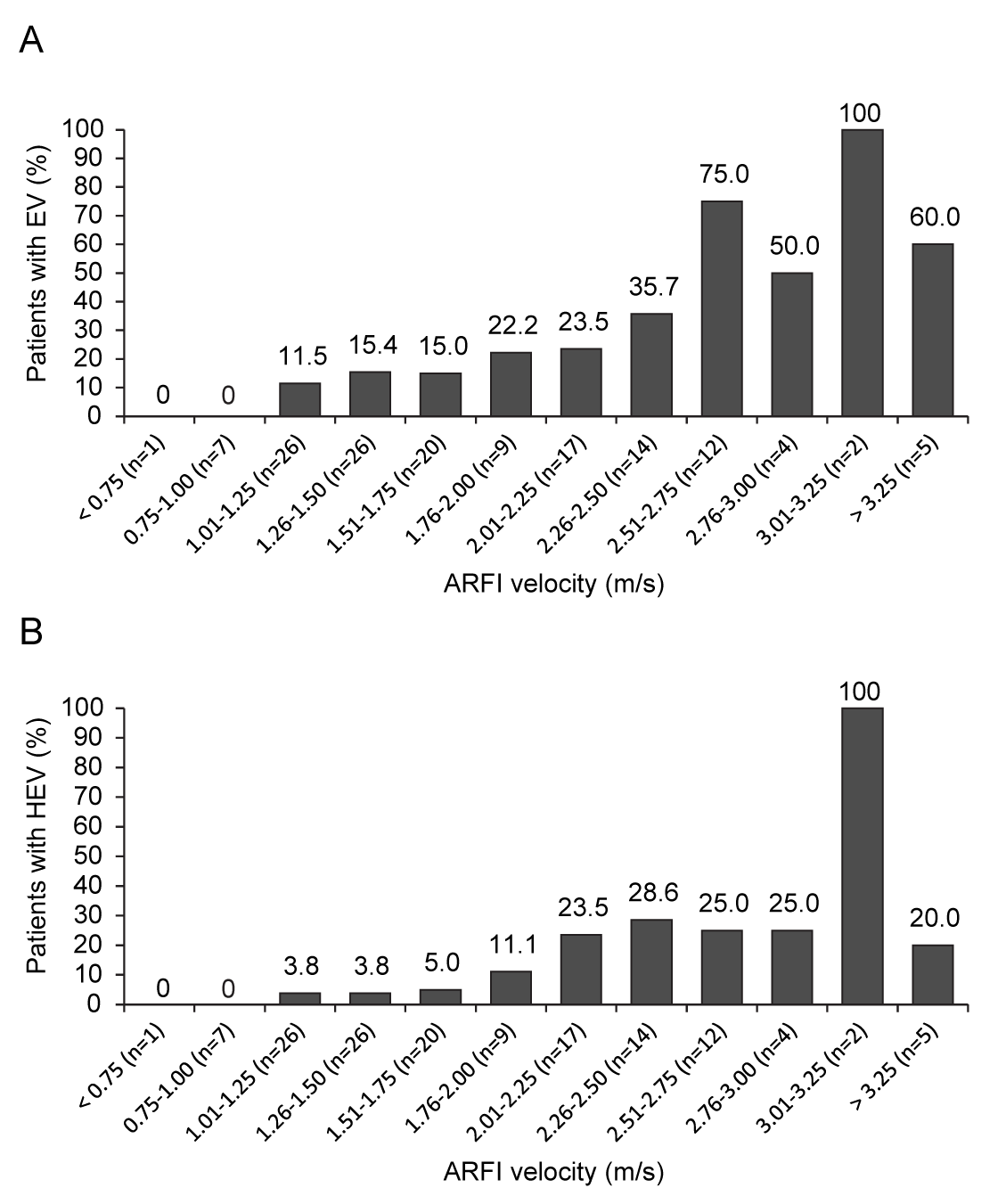

Supplement: S2 Fig — The prevalence of EVs and HEVs tends to increase as ARFI velocity increases. (TIF) [file pone.0121009.s002.tif]

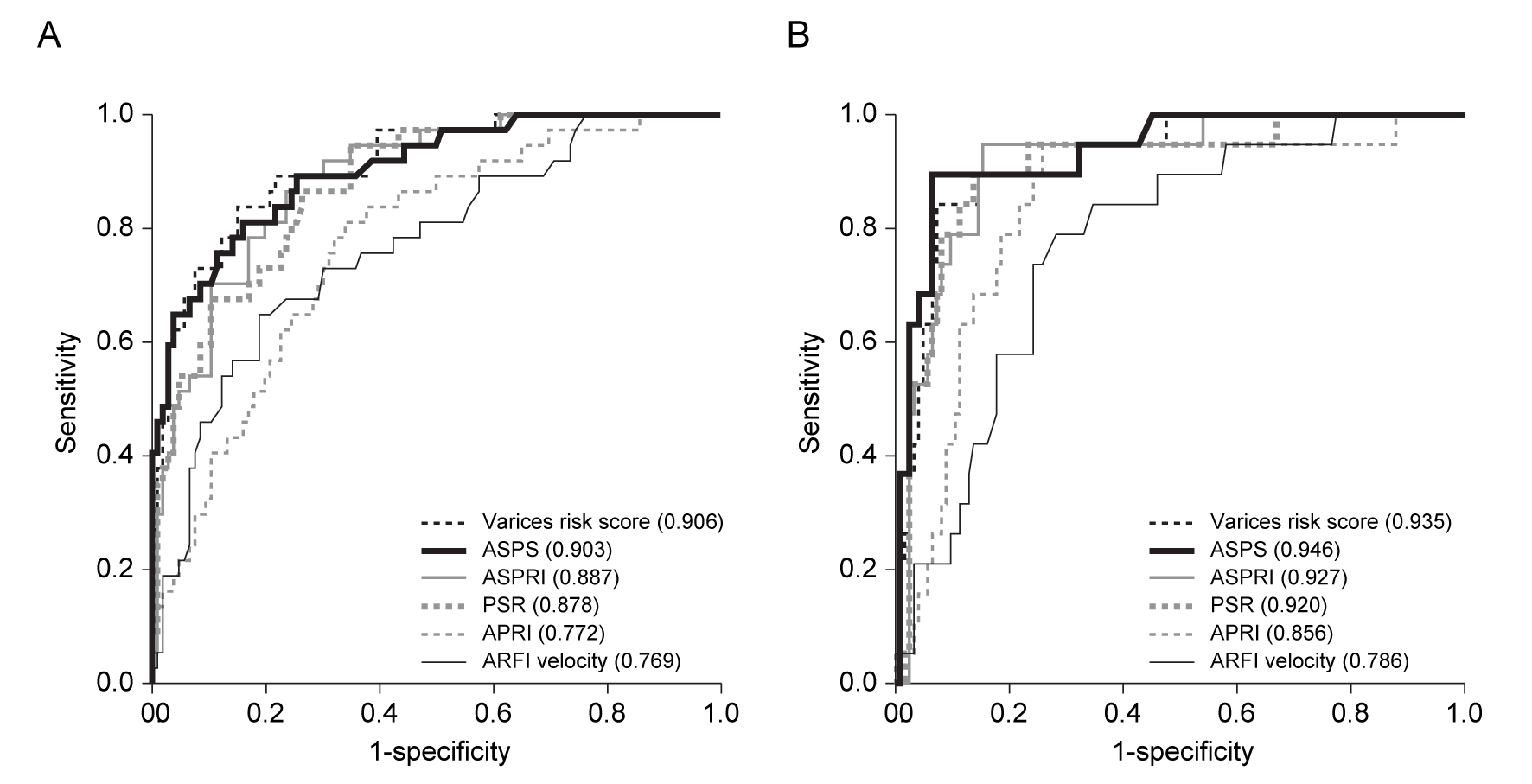

Supplement: S3 Fig — Varices risk score and ASPS showed the best diagnostic performance in predicting EVs and HEVs (AUROC = 0.906 and 0.903 for EVs; 0.935 and 0.946 for HEVs, respectively; all P<0.05). AUROCs are given in brackets after each non-invasive model. ASPS, ARFI-spleen diameter to platelet ratio; ASPRI, age-spleen-to-platelet ratio index; PSR, platelet-spleen ratio; APRI, AST-to-platelet ratio index; ARFI, acoustic radiation force impulse; EVs, esophageal varices; HEV, high-risk esophageal varices (TIF) [file pone.0121009.s003.tif]
