# Supplementary material for: A Novel Model to Predict Esophageal Varices in Patients with Compensated Cirrhosis Using Acoustic Radiation Force Impulse Elastography
Source: PLoS One. 2015 Mar 31;10(3):e0121009. doi: 10.1371/journal.pone.0121009 (PMC4380431; doi:10.1371/journal.pone.0121009)
Supplement: S1 Table — (DOCX) [file pone.0121009.s004.docx]

| **S1 Table. Correlation analysis between ARFI velocity and other variables.** | | |
| --- | --- | --- |
| Variable | Coefficient | *P* value |
| Serum albumin (g/dL) | -0.451 | <0.001 |
| Prothrombin time (%) | -0.333 | <0.001 |
| Aspartate aminotransferase (IU/L) | 0.314 | <0.001 |
| Spleen diameter (cm) | 0.264 | 0.001 |
| Platelet count (x10^9^/L) | -0.245 | 0.003 |
| Total bilirubin (mg/dL) | 0.216 | 0.01 |
| Age (years) | 0.211 | 0.011 |
| Gender | 0.059 | 0.486 |
| Body mass index (kg/m^2^) | -0.022 | 0.798 |
| Alanine aminotransferase (IU/L) | 0.019 | 0.821 |
| ARFI, acoustic radiation force impulse. | | |
