# Supplementary material for: A Novel Model to Predict Esophageal Varices in Patients with Compensated Cirrhosis Using Acoustic Radiation Force Impulse Elastography
Source: PLoS One. 2015 Mar 31;10(3):e0121009. doi: 10.1371/journal.pone.0121009 (PMC4380431; doi:10.1371/journal.pone.0121009)
Supplement: S2 Table — (DOCX) [file pone.0121009.s005.docx]

| **S2 Table. Diagnostic performances of non-invasive models for prediction of EVs and HEVs in the training set among patients with Child-Pugh class A (n=123 patients in the training set).** | | | | | | | | | | |  |  |  |
| --- | --- | --- | --- | --- | --- | --- | --- | --- | --- | --- | --- | --- | --- |
|  | | Method | AUROC (95% CI) | Cutoff value | Sensitivity (%) | Specificity (%) | PPV (%) | NPV (%) | +LR | -LR | Accuracy (%) | |  |
| EVs | | Varices risk score | 0.877 (0.805-0.929) | -0.95 | 76.9 | 84.5 | 57.1 | 93.2 | 4.97 | 0.27 | 82.9 | |  |
|  | | ASPS | 0.877 (0.805-0.929) | 1.65 | 76.9 | 83.5 | 55.6 | 93.1 | 4.66 | 0.28 | 81.3 | |  |
|  | | ASPRI | 0.856 (0.781-0.913) | 10.3 | 84.6 | 74.2 | 46.8 | 94.7 | 3.28 | 0.21 | 75.6 | |  |
|  | | PSR | 0.845 (0.769-0.904) | 1529.2 | 92.3 | 62.9 | 40.0 | 96.8 | 2.49 | 0.12 | 69.1 | |  |
|  | | APRI | 0.753 (0.668-0.827) | 0.74 | 38.0 | 90.4 | 45.5 | 90.9 | 2.29 | 0.40 | 68.3 | |  |
|  | | ARFI | 0.739 (0.653-0.814) | 2.03 | 61.5 | 81.4 | 47.1 | 88.8 | 3.32 | 0.47 | 77.2 | |  |
| HEVs | | Varices risk score | 0.926 (0.864-0.965) | 0.01 | 90.9 | 90.2 | 47.6 | 99.0 | 9.26 | 0.10 | 90.2 | |  |
|  | | ASPS | 0.937 (0.879-0.973) | 2.78 | 90.9 | 94.6 | 62.5 | 99.1 | 16.97 | 0.10 | 93.5 | |  |
|  | | ASPRI | 0.902 (0.835-0.948) | 13.4 | 90.9 | 85.7 | 38.5 | 99.0 | 6.36 | 0.11 | 86.2 | |  |
|  | | PSR | 0.895 (0.827-0.943) | 855.0 | 90.9 | 89.3 | 45.5 | 99.0 | 8.48 | 0.10 | 89.4 | |  |
|  | | APRI | 0.860 (0.786-0.916) | 1.34 | 90.9 | 88.4 | 43.5 | 99.0 | 7.83 | 0.10 | 87.8 | |  |
|  | | ARFI | 0.775 (0.691-0.845) | 2.03 | 81.8 | 77.7 | 26.5 | 97.8 | 3.67 | 0.23 | 78.0 | |  |
| PPV, positive predictive value; NPV, negative predictive value; LR, likelihood ratio; EVs, esophageal varices; HEVs, high-risk esophageal varices; ASPS, ARFI-spleen diameter to platelet ratio; ASPRI, age-spleen-to-platelet ratio index; PSR, platelet-spleen ratio; APRI, AST-to-platelet ratio index; ARFI, acoustic radiation force impulse.  **P* value is for comparison with ARFI. | | | | | | | | | | | | |  |
