# Supplementary material for: A Novel Model to Predict Esophageal Varices in Patients with Compensated Cirrhosis Using Acoustic Radiation Force Impulse Elastography
Source: PLoS One. 2015 Mar 31;10(3):e0121009. doi: 10.1371/journal.pone.0121009 (PMC4380431; doi:10.1371/journal.pone.0121009)
Supplement: S3 Table — (DOCX) [file pone.0121009.s006.docx]

| **S3 Table. Diagnostic performances of ASPS for prediction of EVs and HEVs in the training and validation sets using dichotomic cutoffs in the entire population and subgroup with HBV.** | | | | | | |
| --- | --- | --- | --- | --- | --- | --- |
| Diagnostic indices | Training set (n = 143) | |  | Validation set (n = 148) | | |
|  | EV (n = 37) | HEV (n = 20) |  | EV (n = 51) | | HEV (n = 30) |
| Cutoff values Sensitivity Specificity PPV NPV +LR -LR AUROC Well classified | 1.67 81.1% (30/37) 84.0% (89/106) 63.8% (30/47) 92.7% (89/96) 5.06  0.23 0.903 83.2% (119/143) | 2.83 90.0% (18/20) 94.3% (116/123) 72.0% (18/25) 98.3% (116/118) 15.81 0.11 0.946 93.7% (134/143) |  | 1.67 92.2% (47/51) 78.4% (76/97) 69.1% (47/68) 95.0% (76/80) 4.26  0.10  0.878 83.1% (123/148) | | 2.83 66.7% (20/30) 78.8% (93/118) 44.4% (20/45) 90.3% (93/103) 3.15  0.42  0.814 76.4% (113/148) |
| Subgroup with HBV | Training set (n = 88) | |  | Validation set (n = 91) | | |
|  | EV (n = 25) | HEV (n = 15) |  | EV (n = 28) | HEV (n = 12) | |
| Cutoff values Sensitivity Specificity PPV NPV +LR -LR AUROC Well classified | 2.42 68.0% (17/25) 93.7% (59/63) 81.0% (17/21) 88.1% (59/67) 10.7  0.34 0.868 86.4% (76/88) | 2.83 86.7% (13/15) 95.9% (70/73) 81.2% (13/16) 97.2% (70/72) 21.1 0.14 0.930 94.3% (83/88) |  | 1.59 89.3% (25/28) 79.4% (50/63) 65.8% (25/38) 94.3% (50/53) 4.33  0.13  0.863 82.4% (75/91) | 3.57 83.3% (10/12) 78.5% (62/79) 37.0% (10/27) 96.9% (62/64) 3.87  0.21  0.838 79.1% (72/91) | |
| ASPS, ARFI-spleen diameter to platelet ratio; EVs, esophageal varices; HEVs, high-risk esophageal varices; PPV, positive predictive value; NPV, negative predictive value; LR, likelihood ratio; AUROC, area under the receiver operating characteristic curve; HBV, hepatitis B virus. | | | | | | |
