# Supplementary material for: A Novel Model to Predict Esophageal Varices in Patients with Compensated Cirrhosis Using Acoustic Radiation Force Impulse Elastography
Source: PLoS One. 2015 Mar 31;10(3):e0121009. doi: 10.1371/journal.pone.0121009 (PMC4380431; doi:10.1371/journal.pone.0121009)
Supplement: S4 Table — (DOCX) [file pone.0121009.s007.docx]

| **S4 Table.** **Diagnostic performances of ASPS cutoffs for prediction of HEVs in the training and validation sets.** | | | | | |
| --- | --- | --- | --- | --- | --- |
|  |  | ≥ 95% sensitivity | ≥ 95% specificity | ≥ 95% NPV | ≥ 95% PPV |
| Training set (n=143) | Cutoff Sensitivity Specificity PPV NPV +LR -LR Well classified | 1.35 95.0 (75.1 - 99.9) 68.3 (59.3 – 76.4)  32.8 (20.9 - 46.5)  98.8 (93.6 - 100.0) 3.00 (2.3 – 4.0)  0.07 (0.01 - 0.5)  72.0% (103/143) | 3.83 70.0 (45.7 - 88.1) 96.8 (91.9 - 99.1) 77.8 (51.5 - 93.9) 95.2 (89.8 - 98.2) 21.53 (7.9 - 58.8) 0.31 (0.2 - 0.6) 92.3% (132/143) | 2.83 90.0 (68.3 - 98.8) 94.3 (88.6 - 97.7) 72.0 (50.1 - 88.2) 98.3 (94.0 - 99.8) 15.81 (7.6 - 33.0) 0.11 (0.03 - 0.4) 93.7% (134/143) | 5.28 40.0 (19.1 – 63.9) 100.0 (97.0 - 100.0) 100.0 (63.1 – 100.0) 91.1 (85.0 - 95.3) NA 0.60 (0.4 - 0.9) 91.6 % (131/143) |
| Validation set (n=148) | Well classified | 66.2% (98/148) | 76.4% (113/148) | 76.4% (113/148) | 81.8% (121/148) |
| Overall (n=291) | Well classified | 69.1% (201/291) | 84.2% (245/291) | 84.9% (247/291) | 86.6% (252/291) |
| ASPS, ARFI-spleen diameter to platelet ratio; EVs, esophageal varices; HEVs, high-risk esophageal varices; PPV, positive predictive value; NPV, negative predictive value; LR, likelihood ratio; NA, non-analyzable | | | | | |
