# Supplementary material for: A Novel Model to Predict Esophageal Varices in Patients with Compensated Cirrhosis Using Acoustic Radiation Force Impulse Elastography
Source: PLoS One. 2015 Mar 31;10(3):e0121009. doi: 10.1371/journal.pone.0121009 (PMC4380431; doi:10.1371/journal.pone.0121009)
Supplement: S5 Table — Performance of the use of two cutoffs (one to rule out EVs, and one to rule in EVs). (DOCX) [file pone.0121009.s008.docx]

| **S5 Table.** **Diagnostic performances of the chosen cutoffs of ASPS for predicting HEVs. Performance of the use of two cutoffs (one to rule out EVs, and one to rule in EVs).** | | | | |
| --- | --- | --- | --- | --- |
| Considered as not having HEVs | Considered as indeterminate | Considered as having HEVs | Total misclassified | Total well classified |
| < 2.83 TS = 118/143 VS = 103/148 Misclassified TS: 2 Misclassified VS: 10 | 2.83~5.28 TS = 17/143 = 11.9% VS = 30/148 = 20.3% HEVs TS: 10 HEVs VS: 11 | > 5.28 TS = 8/143 VS = 15/148 Misclassified TS: 0 Misclassified VS: 6 | TS: 2/143 (1.4%) VS: 16/148 (10.8%) | TS: 124/143 (86.7%) VS: 102/148 (68.9%) |

TS, training set; VS, validation set.
